# Concerted action of Aurora B, Polo and NHK-1 kinases in centromere-specific histone 2A phosphorylation

**DOI:** 10.1016/j.yexcr.2007.04.038

**Published:** 2007-05-25

**Authors:** Amy L. Brittle, Yasuaki Nanba, Takashi Ito, Hiroyuki Ohkura

**Affiliations:** a The Wellcome Trust Centre for Cell Biology and Institute of Cell Biology, School of Biological Sciences, The University of Edinburgh, Edinburgh EH9 3JR, UK; b Department of Biochemistry, Nagasaki University School of Medicine, Nagasaki, Nagasaki 852-8523, Japan

**Keywords:** Histone, Kinase, NHK-1, Aurora, Polo, Cyclin, Centromere, *Drosophila*

## Abstract

The spatial and temporal control of histone modifications is crucial for precise regulation of chromatin structure and function. Here we report that phosphorylation of H2A at threonine 119 (T119) is enriched at centromere regions in *Drosophila* mitosis. We found that the Aurora B kinase complex is essential for this phosphorylation at centromeres, while Polo kinase is required to down-regulate H2A phosphorylation on chromosome arms in mitosis. Cyclin B degradation triggers loss of centromeric H2A phosphorylation at anaphase onset. Epistasis analysis indicated that Polo functions upstream of the H2A kinase NHK-1 but parallel to Aurora B. Therefore, multiple mitotic kinases work together to specify the spatial and temporal pattern of H2A T119 phosphorylation.

## Introduction

In eukaryotes, genomic DNA is first packaged into nucleosomes and then organised into higher-order chromatin structures. Chromatin organisation is locally or globally changed in response to external and internal signals. The changes are required for executing critical biological functions, most notably in regulated gene expression and chromosome segregation. Various post-translational modifications take place on histones, mostly in their tail domains, and play critical roles in the regulation of chromatin structure and function, either directly or indirectly through the recruitment of specific chromatin binding proteins [1]. The importance of histone modifications in gene expression is well appreciated and has led to the hypothesis of ‘the histone code’, which proposes that the combination of various histone modifications defines the pattern of gene expression [2].

Upon entry into mitosis, chromatin undergoes dramatic morphological changes to form mitotic chromosomes. On mitotic chromosomes, centromeres form unique chromosomal domains which are crucial for chromosome segregation in two respects [3]. First, centromeres are sites which connect two sister chromatids through cohesins until anaphase. Second, they serve as the foundation for kinetochores which provide the sites for microtubule attachment. To execute these functions, centromeres need to adopt a specialised chromatin structure which also changes during the cell cycle, particularly at the entry into mitosis, at the metaphase–anaphase transition and during exit from mitosis. Moreover, different regulation is also required for meiotic divisions to achieve a correct meiotic chromosome segregation pattern [4].

Recently a novel phosphorylation site was identified at threonine 119 (T119) in the C-terminal tail of *Drosophila* H2A [5]. The site is conserved in H2A amongst eukaryotes (serine in yeasts), but not in H2A variants, such as H2Av and H2AX. Here we show H2A T119 phosphorylation is enriched at centromeres during *Drosophila* mitosis. The Aurora B complex is required for this phosphorylation in centromeric regions, while Polo kinase suppresses phosphorylation by NHK-1 on chromosome arms. Inactivation of Cdc2 kinase is required for loss of centromeric phosphorylation at the metaphase-anaphase transition. Therefore, these mitotic kinases together control the temporal and spatial pattern of H2A phosphorylation at centromeres.

## Materials and methods

### Molecular and immunological techniques

Standard immunological, DNA manipulation and protein techniques were followed throughout [6,7]. Mouse α-tubulin antibody DM1A (Sigma) was used as a loading control in western blots. For immunoblotting, peroxidase-conjugated secondary antibodies (Jackson Lab) were used and detected using an ECL kit (Amersham). Primary antibodies used in this study include antibodies against Histone H2A (Upstate), dH2A-pT119 [5], phospho-H3 (Ser10; Upstate), CID [8], α-tubulin (DM1A; Sigma), GFP (3E6; Molecular Probes) and Aurora B [9].

### Immunofluorescence microscopy

Culture and RNAi of S2 cells were carried out as described [10,11]. Effective depletion of target proteins was monitored by immunoblots or appearance of predicted phenotypes. S2 cells were immunostained as described with the exception that cells were fixed with 4% paraformaldehyde in PBS for 5 min [10]. Larval central nervous systems were dissected from late third instar larvae and fixed with 11% formaldehyde in 0.7% NaCl as described [12]. Secondary antibodies conjugated with Cy3 or Alexa488 (Jackson Lab or Molecular Probes) were used at 1/250–1/1000 dilution. S2 cells were transfected using Effectene Transfection Reagent (Qiagen). Non-degradable cyclin B fused to GFP (pUASp-CBTPM-GFP [13]) was co-transfected with ubiquitin-GAL4 to induce expression. Transfected cells were identified by the presence of GFP. The presence of dH2A-pT119 on centromeres of segregated chromosomes (> 50 cells) was scored.

Cultured cells were examined using a Plan-Apochromat objective lense (100×, 1.4NA; Zeiss) attached to an Axioplan2 (Zeiss). Images were captured by a CCD camera (Orca; Hamamatsu) using OpenLab2 (Improvision). Larval central nervous systems were taken using a Plan-Apochromat lense (63×, 1.4NA; Zeiss) attached to an Axiovert 200 M (Zeiss) with a confocal scan head (LSM510meta; Zeiss). Confocal images were presented as a maximum intensity projection of the Z-stacks. All digital images were imported to Photoshop (Adobe) and adjusted for brightness and contrast.

### Phosphatase treatment

For western blotting of phosphatase treated cell extract, cell extracts were obtained by resuspending S2 cells in lysis buffer (150 mM NaCl, 20 mM Tris, 5 mM EDTA, 1% NP-40) with or without phosphatase inhibitors (100 mM NaF, 2 μM okadaic acid, 100 mM β-glycero-phosphate, 15 mM *p*-nitrophenylphosphate) and incubating on ice for 10 min. Lambda phosphatase (NEB) was added to the cell extract without phosphatase inhibitors and both samples incubated for 30 min at 37 °C. 2× SDS sample buffer was then added to the extracts and boiled for 3 min. Samples were then western blotted with anti-dH2A-pT119 to compare phospho-protein levels. In addition, cells immediately resuspended in 1× SDS sample buffer were included for comparison.

For phosphatase treatment of fixed cells for immunofluorescence with the anti-dH2A-pT119 antibody, cells were fixed with 4% paraformaldehyde in PBS followed by incubation with lambda phosphatase for 1 h at 37 °C. Cells were then washed and immunostained as described above. Microscope images with the same exposure settings were taken of immunostained cells with and without phosphatase treatment. Average pixel intensity of dH2A-pT119 staining on the DNA was measured in interphase and mitotic cells (16 cells in 2 separate experiments).

### Fly stocks

Standard techniques for fly manipulation were followed [14]. All stocks were grown at 25 °C in standard cornmeal media. A null *nhk-1* mutant (*nhk-1*^*E107*^) used in this study was previously described [15].

## Results

### H2A T119 phosphorylation is specific to centromeres in mitosis

**Fig. 1:**
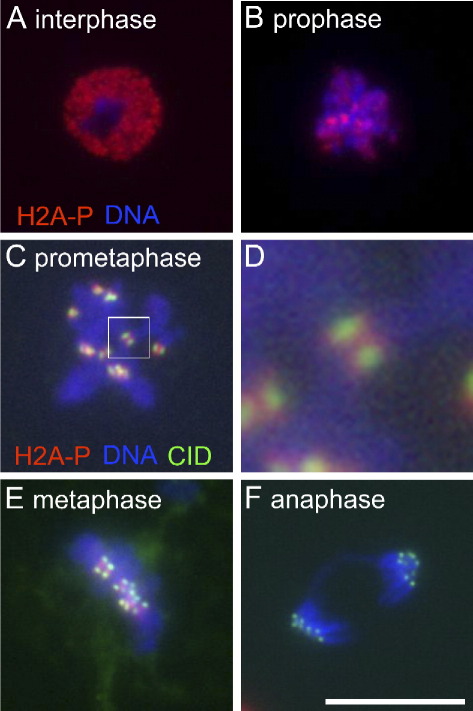
Dynamic change of H2A T119 phosphorylation in the cell cycle. S2 cells were immunostained with anti-dH2A-pT119 antibody. H2A T119 phosphorylation was over all chromatin in interphase (A) but enriched to centromeric regions in prophase (B) and maintained through prometaphase (C) and metaphase (E). The phosphorylation was lost in anaphase (F). The boxed region in C is magnified in D. Scale bar = 10 μm.

To examine the spatial and temporal control of H2A T119 phosphorylation in cells, we immunostained *Drosophila* S2 cells using an antibody which specifically recognises this phosphorylated form of H2A (anti-dH2A-pT119 [5]). We found a dynamic change in the phosphorylation pattern of H2A during the cell cycle. In interphase, phosphorylation was present throughout the chromatin in the nucleus (Fig. 1A). Interestingly, in mitosis, as the chromosomes begin to condense, phosphorylation was no longer spread throughout the chromatin but produced a more punctate pattern (Fig. 1B). Co-staining with a centromeric marker CID (the CENP-A homologue; [8,16]) revealed that in prometaphase and metaphase, phosphorylation was enriched in regions between and surrounding CENP-A positive regions, which we refer to as the centromeric regions (Figs. 1C–E). This phosphorylation became dramatically reduced at the onset of anaphase (Fig. 1F). Phosphorylation only returned on decondensed chromatin at the end of mitosis.

Specificity of the signal obtained by this phospho-H2A antibody was confirmed by treatment with lambda protein phosphatase. Lambda phosphatase treatment of S2 cell extracts eliminated a single band (which comigrates with H2A) recognised by the antibody on immunoblots (Supplementary Fig. 1). Furthermore, the immunofluorescent signals obtained by the phospho-H2A antibody were greatly reduced by lambda phosphatase treatment of fixed S2 cells (Supplementary Fig. 1).

In syncytial embryos and oocytes, entire mitotic/meiotic chromosomes are stained with the anti-dH2ApT119 antibody [5,17]. To confirm that the phosphorylation pattern found in S2 cells is not specific to this cell line, we examined H2A phosphorylation in somatic cells of developing flies. The larval central nervous system (CNS) is the tissue most commonly used for the study of standard mitotic cell cycles, which have two gap phases and checkpoint regulation [18]. Immunostaining of larval CNSs revealed a similar temporal and spatial pattern of H2A T119 phosphorylation as found in S2 cells (Supplementary Fig. 2A).

### Centromeric H2A T119 phosphorylation depends on Aurora B kinase

Previously, the conserved protein kinase NHK-1 was identified as phosphorylating H2A T119 in vitro [5]. A female sterile mutation in *NHK-1* greatly reduced phosphorylation at this site in oocytes, but not in follicle or nurse cells [17]. This indicated that NHK-1 is the major kinase responsible for this phosphorylation at least in the oocyte nucleus. To test whether NHK-1 is responsible for this phosphorylation in S2 cells, we examined whether depletion of this kinase by RNA interference (RNAi) affects the phosphorylation. Down-regulation of NHK-1 in S2 cells did not eliminate the signal of the phospho-H2A antibody in immunostaining (Supplementary Figs. 3 and 5). This result was further confirmed by immunostaining of larval CNSs from a null mutant of *NHK-1* (*nhk1*^*E107*^ [15]) (Supplementary Fig. 2B). These results indicated that either a residual amount of NHK-1 kinase is sufficient to phosphorylate this site or kinases other than NHK-1 can phosphorylate this site in the absence of NHK-1.

**Fig. 2:**
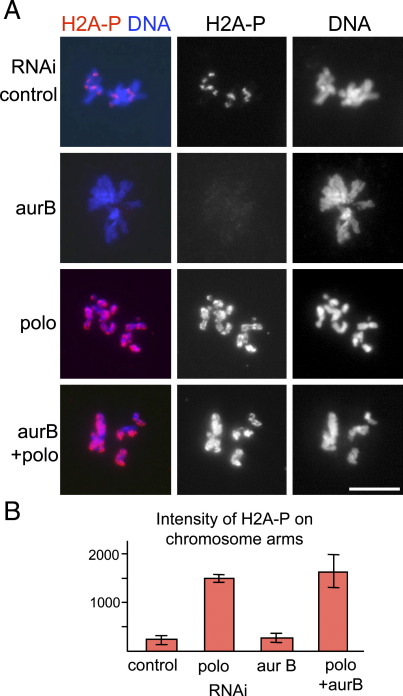
Aurora B and Polo kinases are required for enrichment of H2A T119 phosphorylation at centromeric regions in mitosis. (A) S2 cells were immunostained using anti-dH2A-pT119 antibody after single or double depletion of Aurora B and Polo by RNAi. Aurora B is required for H2A T119 phosphorylation at centromeric regions, while Polo is required for suppressing the phosphorylation on chromosome arms. Scale bar = 10 μm. (B) Average pixel intensity of anti-dH2A-pT119 immunofluorescent signals on chromosome arms. The differences between the control and Polo or Polo/Aurora B are statistically significant (*p* < 0.001), while the differences between the control and Aurora B, or Polo and Polo/Aurora B are not significant (*p* ?>> 0.3). The H2A phosphorylation on chromosome arms in Polo-depleted cells was not dependent on Aurora B.

To identify the regulatory mechanism of this dynamic change in H2A T119 phosphorylation, we first examined the potential role of Aurora B kinase which localises to the same centromeric domain as the H2A phosphorylation [19]. After Aurora B was depleted by RNAi, S2 cells were immunostained with phospho-H2A antibody. In Aurora B-depleted cells, the intense centromeric staining in mitotic cells was reduced to levels equivalent to that on the chromosome arms (Fig. 2A). However, nuclear staining in interphase cells remained high (Supplementary Fig. 5), suggesting that the phosphorylation is regulated in interphase and mitosis by different mechanisms.

Aurora B kinase is part of at least two functionally distinct complexes [19], a core complex (containing INCENP) and a larger complex (additionally containing Survivin and Borealin/DasraB). To understand which complex is required for the H2A phosphorylation, we tested the requirement of other subunits for the phosphorylation. Depletion of any one of INCENP, Survivin and Borealin by RNAi greatly reduced H2A phosphorylation in centromeric regions in mitosis (Supplementary Fig. 4). Interphase phosphorylation was not affected in any of the cases. These results indicated that the large AuroraB complex is required for centromeric phosphorylation of H2A at T119 in mitosis.

### Polo kinase down-regulates the H2A phosphorylation on chromosome arms

To further study the regulatory mechanism of the phosphorylation, we examined the role of the crucial mitotic regulator Polo kinase [20]. After Polo kinase was depleted by RNAi, S2 cells were immunostained with phospho-H2A antibody. Surprisingly, in Polo-depleted cells, H2A T119 phosphorylation was not restricted to centromeric regions in mitosis but remained at a high level on the entire chromosome arms (Fig. 2A). Quantitative analysis indicated that the fluorescent signal from the phospho-H2A antibody on chromosome arms was dramatically increased in the absence of Polo kinase (Fig. 2B). This result suggests that Polo kinase is directly or indirectly required for down-regulating H2A T119 phosphorylation on chromosome arms to enrich the phosphorylation at centromeric regions.

### Polo suppresses phosphorylation by the H2A kinase NHK-1

To identify the relationship between Aurora B and Polo actions, both of the kinases were depleted simultaneously. If a loss of Polo kinase misregulates Aurora B kinase, simultaneous depletion would suppress H2A T119 phosphorylation on chromosome arms. Immunostaining of cells depleted of both Aurora B and Polo showed a high level of phosphorylation on the entire chromosome arms (Fig. 2). This indicated that H2A T119 phosphorylation on chromosome arms induced by loss of Polo kinase was independent of Aurora B activity.

**Fig. 3:**
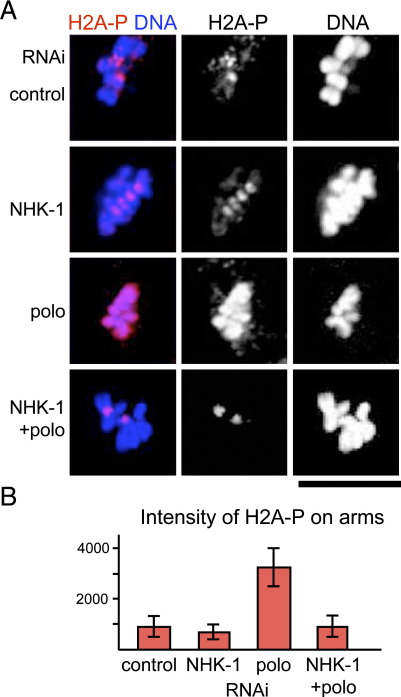
H2A T119 phosphorylation on chromosome arms in the absence of Polo kinase depends on NHK-1 kinase. (A) S2 cells were immunostained using anti-dH2A-pT119 antibody after single or double depletion of NHK-1 and Polo by RNAi. (B) Average pixel intensity of anti-dH2A-pT119 immunofluorescent signals on chromosome arms. The differences between Polo and others are statistically significant (*p* < 0.001), while the differences between the control, NHK-1 and Polo/NHK-1 are not significant (*p* ?>> 0.2). The H2A phosphorylation on chromosome arms in Polo-depleted cells was dependent on NHK-1, which is known to phosphorylate H2A T119.

Next we tested the relationship between Polo and the H2A kinase NHK-1 by co-depletion. We found that NHK-1 depletion suppresses H2A T119 phosphorylation on arms induced by a loss of Polo (Fig. 3). Quantitative analysis confirmed that the phospho-H2A signal on chromosome arms in Polo NHK-1 double depletions was decreased to a level comparable to that of the control or NHK-1 depletion.

Finally we tested the phenotype of double depletion of Aurora B and NHK-1. Like Aurora B single depletion, H2A T119 phosphorylation was greatly reduced from centromeric regions of mitotic chromosomes (Supplementary Fig. 5).

These epistasis studies suggested that Polo functions upstream of NHK-1 to suppress H2A T119 phosphorylation, but is independent of Aurora B.

### Cyclin B degradation triggers a loss of H2A phosphorylation at initiation of anaphase

**Fig. 4:**
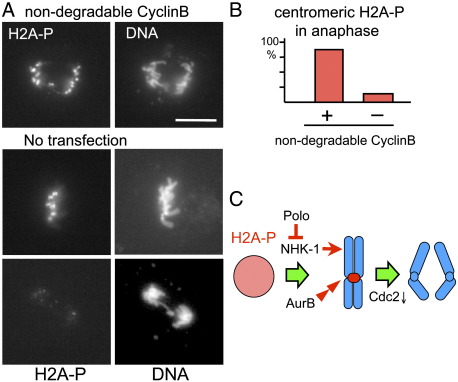
Cyclin B degradation is required for the loss of H2A phosphorylation at the metaphase-anaphase transition. (A) Expression of non-degradable Cyclin B (CyclinB.TPM-GFP) prevented loss of H2A T119 phosphorylation at anaphase. Scale bar = 10 μm. (B) Frequencies of H2A T119 phosphorylation at centromeric regions amongst anaphase cells with or without expression of non-degradable Cyclin B (CyclinB.TPM) tagged with GFP. The difference is statistically significant (*p* < 0.001). (C) A schematic diagram for regulation of H2A phosphorylation at T119 in mitosis. In interphase, phosphorylation is found throughout the chromatin. In early mitosis, the phosphorylation is enriched at centromeric regions. This process is mediated by the concerted action of Aurora B and Polo kinases which are required for H2A T119 phosphorylation at centromeric regions and suppressing the phosphorylation on arms, respectively. At the onset of anaphase, the phosphorylation at centromeric regions is lost. Cyclin B degradation or Cdc2 inactivation is required for the loss.

Centromeric H2A T119 phosphorylation becomes greatly reduced at the onset of anaphase indicating a change in its regulation at this time. After alignment of all chromosomes, APC/Cdc20 triggers degradation of Cyclin B and securin, leading to inactivation of Cdc2 kinase and activation of separase which cleaves cohesin to initiate anaphase [21]. To separate Cyclin B degradation from securin degradation, we expressed non-degradable Cyclin B in S2 cells and examined H2A phosphorylation by immunostaining. As previously reported [22,23,13], expression of non-degradable Cyclin B did not inhibit the onset of anaphase but prevented exit from mitosis, resulting in an accumulation of anaphase cells with overcondensed chromosomes. In cells expressing non-degradable Cyclin B, H2A phosphorylation was still retained at centromeric regions in most anaphase cells (Figs. 4A, B). Therefore, we concluded that cyclin B degradation, not anaphase onset, is required for triggering loss of phosphorylation at the metaphase-anaphase transition.

## Discussion

In this study, we found dynamic changes in H2A T119 phosphorylation during the *Drosophila* cell cycle. This phosphorylation is enriched at centromeric regions early in mitosis and lost at the onset of anaphase. In interphase, H2A T119 phosphorylation was found throughout chromatin. Furthermore, our evidence showed that the combined action of at least four conserved mitotic kinases is required for precise spatial and temporal regulation of H2A T119 phosphorylation (Fig. 4C). Aurora B kinase is required for the enrichment of phosphorylation at centromeric regions in mitosis. Polo kinase is required for suppressing H2A phosphorylation by NHK-1 on chromosome arms. Furthermore, inactivation of Cdc2 kinase induced by Cyclin B degradation is required for the loss of centromeric phosphorylation at the onset of anaphase.

Currently we do not know what the function of this H2A phosphorylation is in cells. In higher eukaryotes which have many copies of histone genes, the function of histone modifications has been studied only indirectly by down-regulating responsible modifying enzymes. Unfortunately this approach is not suitable for kinases as they are likely to have multiple substrates (for example, Cdc2 and Aurora B mediating H1 and H3 phosphorylation).

Centromeric distribution and regulation by conserved mitotic kinases may tempt us to speculate possible involvement of H2A T119 phosphorylation in chromosome segregation in mitosis. The phosphorylation might be important for generating or sensing tension between sister chromatids, or modes of microtubule attachment to kinetochores through the formation of centromere-specific chromatin or recruitment of centromere proteins during mitosis. A loss or misregulation of the H2A phosphorylation may be responsible for a subset of the highly pleiotropic phenotypes observed after down-regulation of Aurora B or Polo [19,20]. It would be a future challenge to define the precise roles of this H2A phosphorylation.
