# Supplementary material for: Concerted action of Aurora B, Polo and NHK-1 kinases in centromere-specific histone 2A phosphorylation
Source: Exp Cell Res. 2007 May 25;313(13):2780–5. doi: 10.1016/j.yexcr.2007.04.038 (PMC2131725; doi:10.1016/j.yexcr.2007.04.038)
Supplement: Supplementary Fig. 1 [file mmc1.pdf]

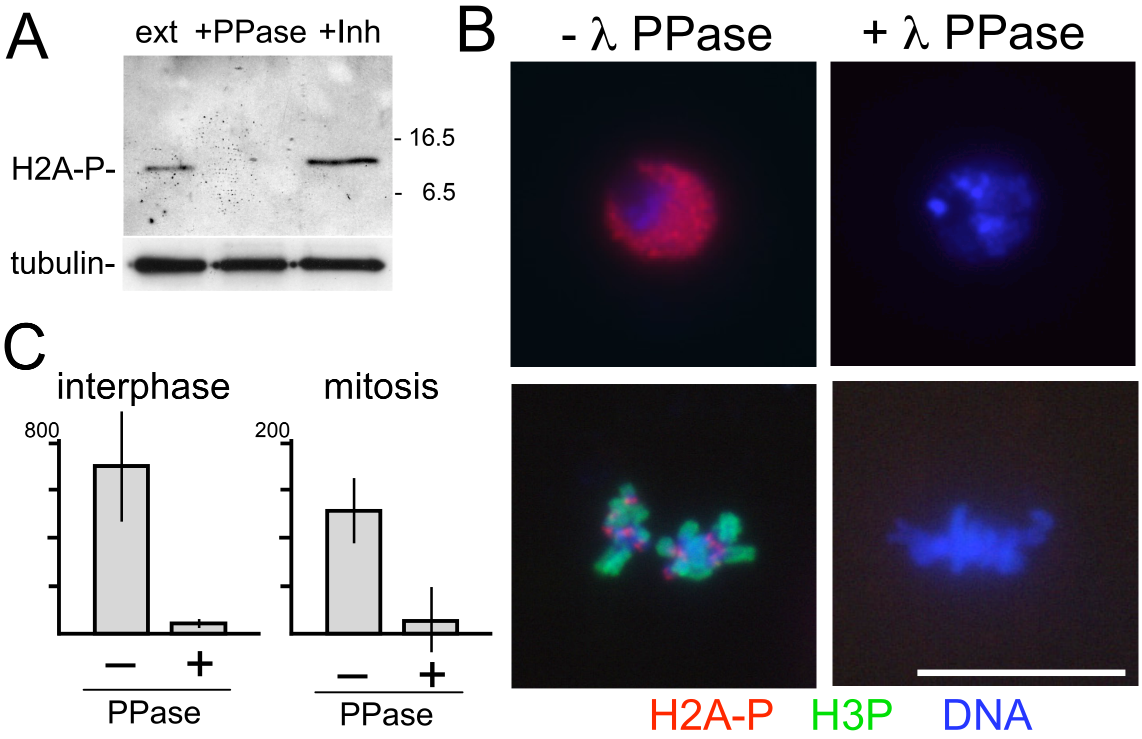

Supplementary Figure 1. Anti-dH2ApT119 antibody specifically recognises a phosphorylated form. (A) Following λ-protein phosphatase treatment a band with the size of H2A was no longer recognised by the anti-dH2ApT119 antibody. S2 cell extract was either boiled immediately with sample buffer (ext) or incubated for 10 minutes in the presence of lambda phosphatase (+PPase) or phosphatase inhibitors (+Inh). (B) Specific staining with H2ApT119 staining was removed by phosphatase treatment. Fixed S2 cells were treated with or without lambda phosphatase followed by immunofluorescence with anti-dH2ApT119 (red), anti-phospho H3 (S10; green) and DNA (blue). H2ApT119 staining is lost from interphase (upper panel) and mitotic (lower panel) chromosomes following phosphatase treatment. Bar=10μm. (C) The level of H2A pT119 staining was quantified by measuring the mean pixel intensity around the DNA in interphase and pre-anaphase mitosis (16 interphase and mitotic cells were measured). Standard deviations are represented by vertical lines.

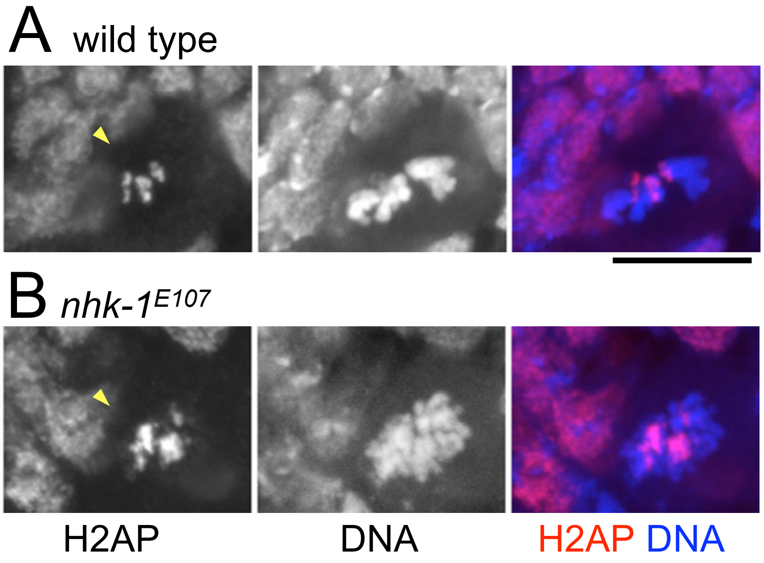

Supplementary Figure 2. H2A T119 phosphorylation in larval central nervous systems. Larval central nervous systems dissected from wild type (A) and a null *nhk-1* mutant (*nhk-1^{E107}*; B; Cullen et al., 2005) were fixed and immunostained with anti-dH2ApT119 antibody and DAPI. Mitotic neuroblasts (arrowheads) were surrounded by smaller interphase cells. H2ApT119 was distributed in a similar manner as in S2 cells, and not affected by the *nhk-1* mutation. Cell division in the mutant is likely to be supported by residual activity of maternal wild-type NHK-1. The H2A phosphorylation in the mutant could be due to the maternal contribution or the presence of other kinases which can phosphorylate this site. Bar=10μm.

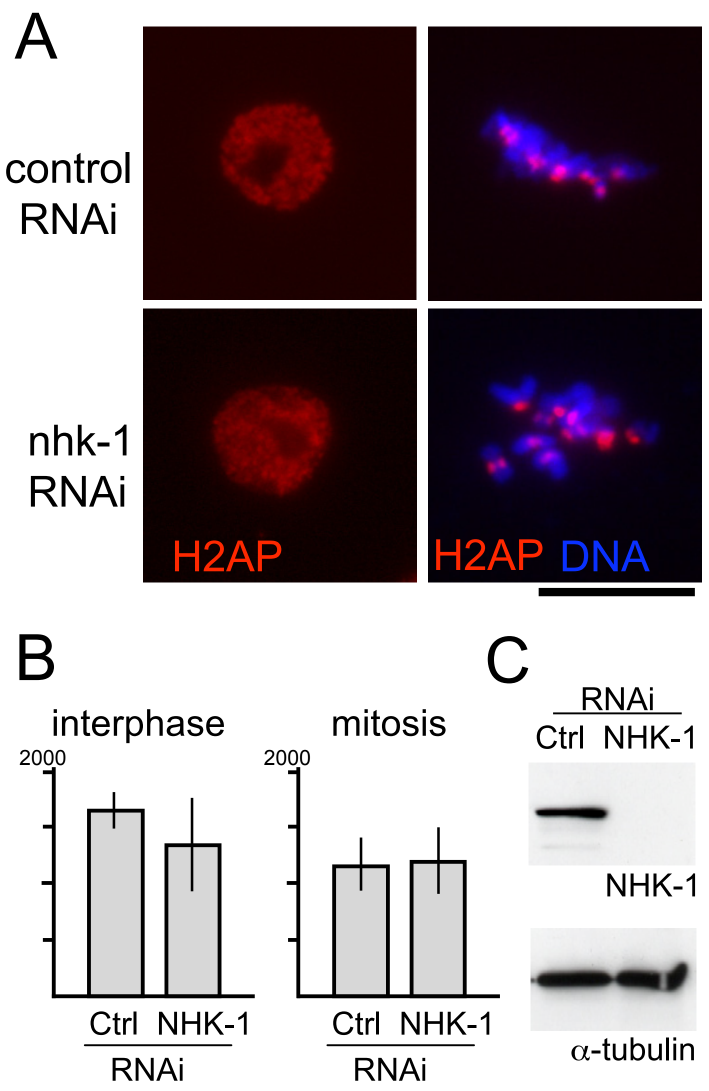

Supplementary Figure 3. NHK-1 down-regulation does not abolish H2ApT119. (A) S2 cells were fixed and immunostained for H2ApT119 after incubation with control or *nhk-1* dsRNA for 7 days. Depletion of NHK-1 was confirmed by immunoblots. Bar=10μm. (B) Average intensity of anti-dH2ApT119 fluorescent signals in interphase nucleus and centromere regions in mitosis. Standard deviations are represented by vertical lines. (C) Immunoblots with NHK-1 and α-tubulin antibodies after RNAi.

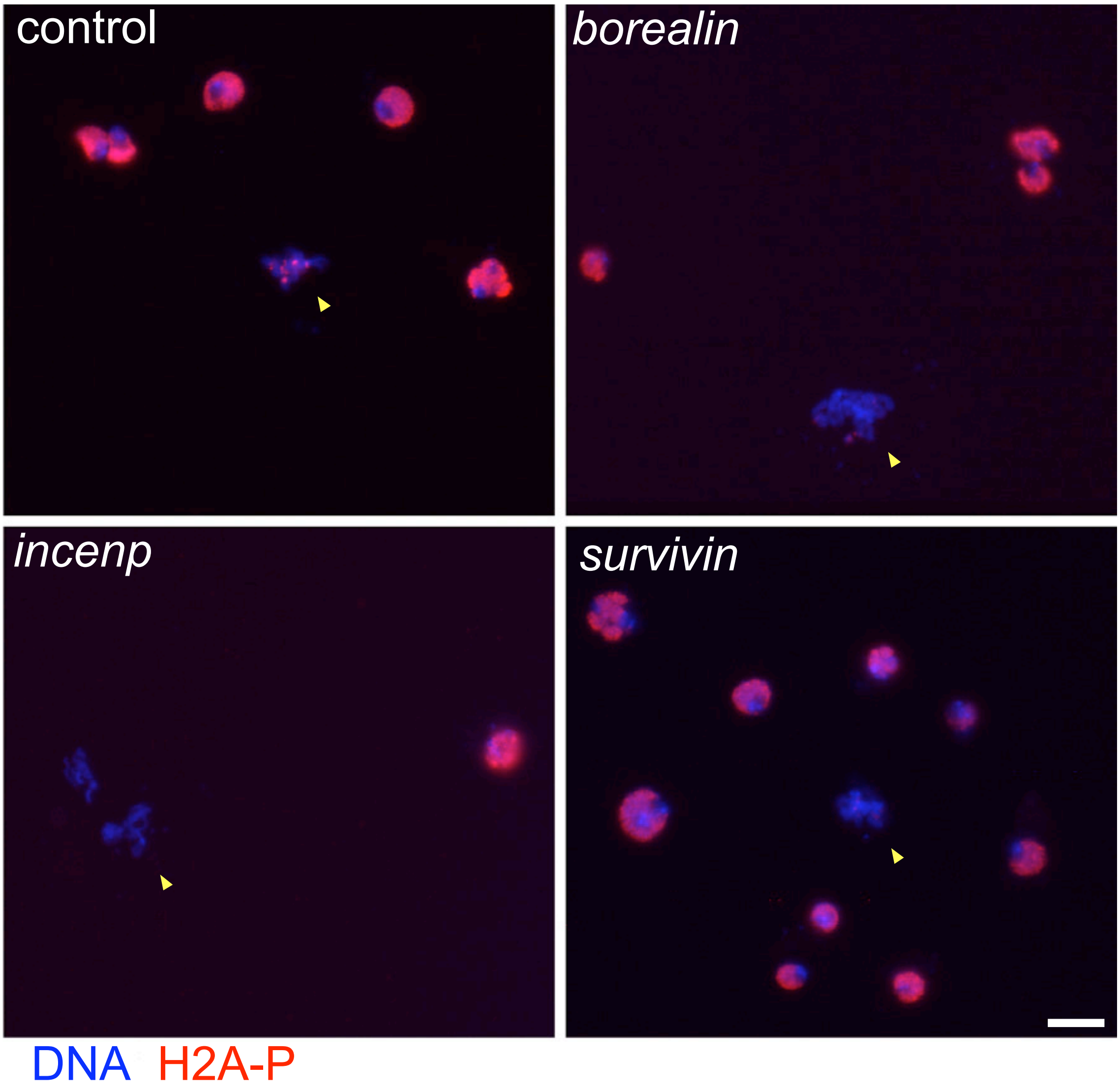

Supplementary Figure 4. Depletion of Aurora B subunits reduces H2ApT119 in mitosis. S2 cells were fixed and immunostained with anti-dH2ApT119 and DAPI after incubation with dsRNA. Arrowheads indicate mitotic cells. Bar=10μm.

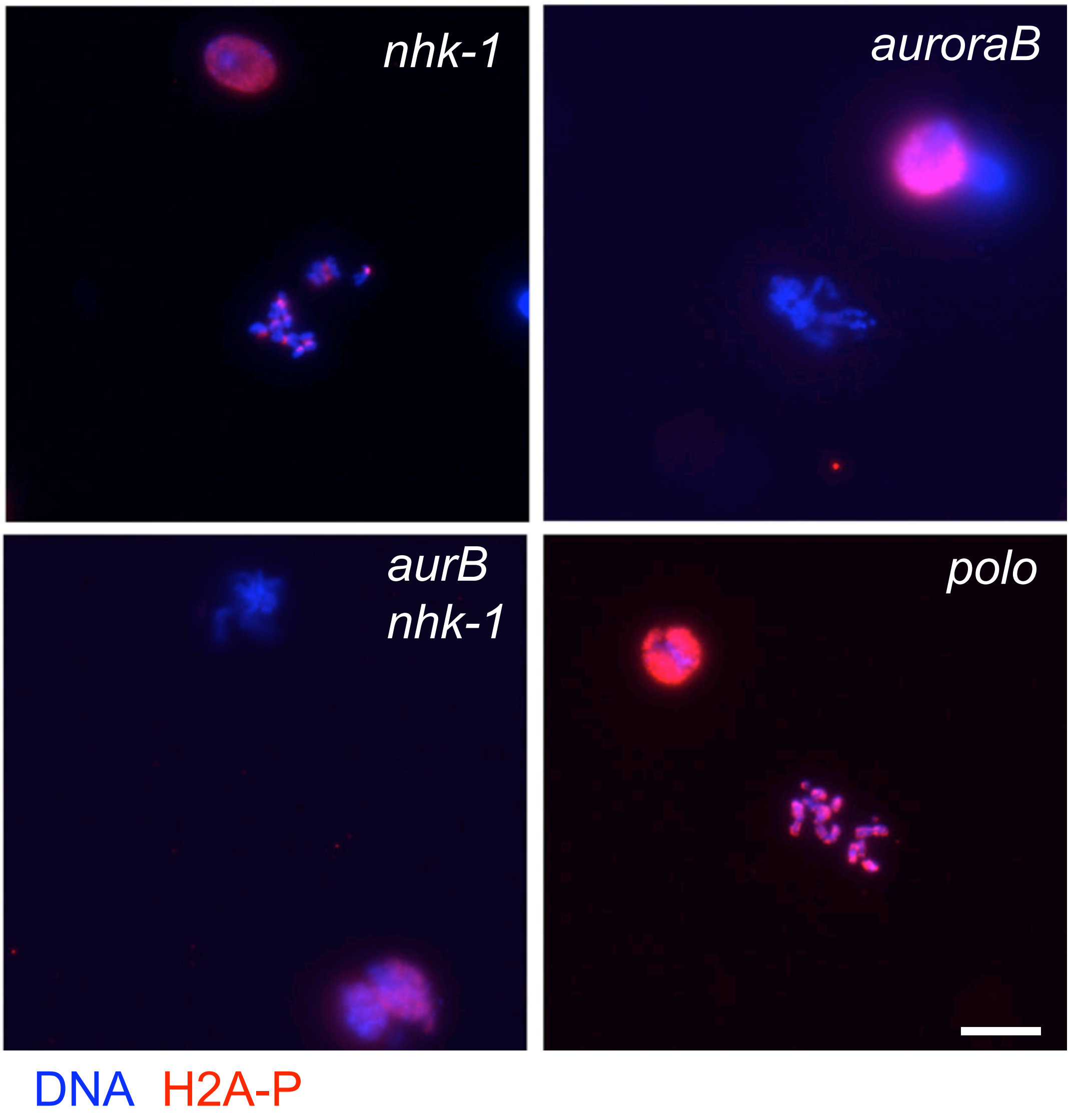

Supplementary Figure 5. Effects of down-regulation of mitotic kinases on H2ApT119. S2 cells were fixed and immunostained with anti-dH2ApT119 and DAPI after incubation with dsRNA. Bar=10μm.
